# Copeptin, a surrogate marker for arginine vasopressin, is associated with cardiovascular risk in patients with polycystic ovary syndrome

**DOI:** 10.1186/1757-2215-7-31

**Published:** 2014-03-14

**Authors:** Basak Karbek, Mustafa Ozbek, Melia Karakose, Oya Topaloglu, Nujen Colak Bozkurt, Evrim Cakır, Muyesser Sayki Aslan, Tuncay Delibasi

**Affiliations:** 1 Department of Endocrinology and Metabolic Diseases, Gaziantep Dr. Ersin Arslan Hospital, Milli Egemenlik bulvarı, Sanlılar Apt, no:51 daire:9 Şehitkamil/Gaziantep, Gaziantep, Turkey; 2 Department of Endocrinology and Metabolic Diseases, Dışkapı Yıldırım Beyazıt Teaching and Research hospital, Ankara, Turkey; 3 Department of Endocrinology and Metabolic Diseases, Amasya Sabuncuoglu Serefettin Training and Research Hospital, Amasya, Turkey

**Keywords:** Polycystic ovary syndrome, Copeptin, Carotid intima media thickness, Insulin resistance, Cardiovascular disease risk

## Abstract

**Background:**

Women with polycystic ovary syndrome (PCOS) have higher risk for cardiovascular disease (CVD). Copeptin has been found to be predictive for myocardial ischemia. We tested whether copeptin is the predictor for CVD in PCOS patients, who have an increased risk of cardiovascular disease.

**Methods:**

This was a cross sectional controlled study conducted in a training and research hospital. The study population consisted of 40 reproductive-age PCOS women and 43 control subjects. We evaluated anthropometric and metabolic parameters, carotid intima media thickness and copeptin levels in both PCOS patients and control group.

**Results:**

Mean fasting insulin, homeostasis model assessment insulin resistance index (HOMA-IR), triglyceride, total cholesterol, low density lipoprotein cholesterol (LDL-C), free testosterone, 17-OH progesterone, Dehydroepiandrosterone sulfate (DHEAS), carotid intima media thickness (CIMT) levels were significantly higher in PCOS patients. Mean copeptin level was in 12.61 ± 3.05 pmol/L in PCOS patients while mean copeptin level was 9.60 ± 2.80 pmol/L in healthy control women (p < 0.001). After adjustment for age and BMI, copeptin level was positive correlated with fasting insulin, free testosterone levels, CIMT, and HOM A-IR.

**Conclusions:**

Copeptin appeared to have an important role in metabolic response and subsequent development of atherosclerosis in insulin resistant, hyperandrogenemic PCOS patients.

## Introduction

Polycystic ovary syndrome is a common endocrine disorder affecting at least 5 to 10% of women of reproductive age [1]. Polycystic ovary syndrome is characterized by hyperandrogenism, menstrual disturbance, anovulation, infertility and obesity [2], and also associated with an increased number of cardiovascular risk factors [3], and early atherosclerosis [4,5]. Hyperinsulinism and insulin resistance are frequent findings in PCOS patients, and these traits have cause-consequence relationships with low-grade chronic inflammation [6], and increased cardiovascular disease risk [7].

Arginine vasopressin (AVP), which is also called antidiuretic hormone, is released from the neurohypophysis as a response to increased plasma osmolality and decreased blood volume. AVP exerts an antidiuretic effect in the kidney and a vasoconstrictive and blood platelet aggregating effect in the vessels. In addition, animal studies have shown effects of AVP on glucose metabolism. AVP influences gluconeogenesis and glycogenolysis in the liver [8,9], insulin and glucagon release by the Langerhans islets of the pancreas [10] and adrenocorticotrophic hormone release from the anterior hypophysis [11]. Vasopressin is a short-lived peptide and most assays have relatively limited sensitivity. An assay has been developed to measure plasma copeptin (copeptin), the C-terminal portion of the precursor of AVP. Copeptin is considered to be a reliable and clinically useful surrogate marker for AVP [12]. In healthy populations and in patients with various cardiovascular diseases, there is a significant positive association between copeptin and AVP levels. However, the association between copeptin and patients with PCOS remains unknown. The present study was, therefore, undertaken to investigate the correlations between copeptin, and the progression of atherosclerosis in PCOS patients.

## Subjects, materials and methods

We studied 40 patients with PCOS and age- body mass index (BMI) matched 43 healthy controls consisting of women with regular ovulatory cycles and normal androgen levels. All patients gave a written consent. All patients were female and nonsmokers. Participants recruited from Turkey, ethnicity of the participants’ is Caucasian (Europe and Middle East). The diagnosis of PCOS was made according to the Rotterdam European Society for Human reproduction and Embryology/American Society for Reproductive Medicine–sponsored PCOS Consensus Workshop Group [13]. The revised diagnostic criteria of PCOS is as follows, with at least two of the following being required;

1. Oligo and/or anovulation that is menstrual disturbance

2. Clinical and/or biochemical signs of hyperandrogenism

3. Polycystic ovarian appearance on ultrasound

Participants who had smoking history, diabetes mellitus, hyperprolactinemia, congenital adrenal hyperplasia, androgen-secreting tumours, thyroid disorders, Cushing syndrome (1 mg dexamethasone suppression test), infection diseases, hypertension, hepatic or renal dysfunction were excluded from the study. Patients were also excluded if they had used within 3 months before enrollment confounding medications, including oral contraceptive agents, antilipidemic drugs, hypertensive medications, and insulin-sensitizing drugs.

Control group (n = 43) consisted of healthy patients who were admitted to check-up unit without any systemic disorder. All of the women in the control group had hirsutism score <8. All women in the control group had regular menses, every 21–35 days. None of the women in the control group had polycystic ovary in ultrasound.

Weight and height were measured in light clothing without shoes. BMI was calculated, dividing the weight divided by square of height (kg/m^2^). Waist circumference was measured at the narrowest level between the costal margin and iliac crest, and the hip circumference was measured at the widest level over the buttocks while the subjects were standing and breathing normally. The waist-to-hip ratio (WHR) was calculated.

The degree of hirsutism was determined by Ferriman-Gallwey (FG) scoring [14]. The BMI, WHR and hirsutism scores were assessed by a single investigator for all of the subjects.

### Measurement of carotid intima media thickness

Carotid intima media thickness (CIMT) was derived from noninvasive ultrasound of the common carotid arteries, using a high-resolution ultrasound machine (Sonoline G 40, Siemens) with 7.5 MHz mechanical sector transducer. The intima media thickness was defined as the distance between the blood-intima and media-adventitia boundaries on B-mode imaging. All scans and image measurements were carried out by the same investigator, who was blinded to the diagnosis of the participants.

#### Biochemical evaluation

Venous blood samples were obtained in the follicular phase of a spontaneous or progesterone induced menstrual cycle. Before the study, blood samples were drawn from each patient after 12 h overnight fasting for the determination of hormones, lipid profile, high-sensitive C- reactive protein (hs-CRP), insulin levels, glucose levels.

Plasma glucose was determined with glucose oxidase/peroxidase method (Gordion Diagnostic, Ankara, Turkey). Serum levels of follicle-stimulating hormone (FSH), luteinizing hormone (LH), prolactin, dehydroepiandrosterone sulfate (DHEAS), total testosterone (T), insulin and thyroid stimulating hormone (TSH) were measured with specific electrochemiluminescence immunoassays (Elecsys 2010 Cobas, Roche Diagnostics, Mannheim, Germany). Serum 17 hydroxyprogesterone was measured by radioimmunoassay. Levels of total-cholesterol, high density lipoprotein cholesterol (HDL-C), and triglyceride (TG) were determined with enzymatic colorimetric assays by spectrophotometry (BioSystems S.A., Barcelona, Spain). Low density lipoprotein cholesterol (LDL-C) was calculated using the Friedewald formula.

Serum hs-CRP was determined using high-sensitive CRP immunonephelometry (BN, Dade-Behring; Marburg, Germany). The cut off for hsCRP was taken 1.5 [15].

Insulin resistance was calculated by using the homeostasis model assessment insulin resistance index (HOMA-IR) [16], according to the formula, fasting plasma glucose (mmol/L) x fasting serum insulin (mU/mL) /22.5. The cut off value was taken 2.7 for HOMA-IR [17].

#### Copeptin

Blood samples collected into the tubes which contain EDTA. The blood centrifuged at 1.600 × g for 15 minutes, the plasma was separated and stored at -80°C until assessment of copeptin. Measurements of copeptin were performed in an EPOCH system (BioTek Instruments, Inc, USA) using the commercially available enzyme-linked immunosorbent assay (ELISA) kit (Phoenix Pharmaceuticals, California, USA) in accordance with the manufactures’ instructions. The assay range of the copeptin, ELISA kit was 0-100 pmol/L. Copeptin levels were expressed as ng/ml. The samples were carried out together in the same experiment.

#### Statistical analyses

Collected data was entered to SPSS version 17. Continuous data were shown as mean ± SD. Chi-squared tests were used to compare differences in rates. Normally distributed variables were compared by using Student *T* test. Data that were not normally distributed, as determined using Kolmogorov–Smirnov test, were logarithmically transformed before analysis. Data are expressed as mean ± SD or median with interquartile range as appropriate. Degree of association between continuous variables was calculated by Pearson correlation coefficient, nonnormally distributed variables was evaluated by spearman’s rho correlation coefficient. The multiple linear regression enter method was used to determine the independent predictors. p value lower than 0.05 was accepted as statistically significant.

## Results

Clinical and endocrinological parameters screened in patients with PCOS and in healthy control subjects were shown in Table 1. We studied 40 PCOS patients (22.97 ± 5.18 years; BMI; 24.40 ± 5.82 kg /m^2^) and 43 age and BMI matched healthy control group (23.63 ± 4.60 years, BMI; 25.44 ± 4.82 kg/m^2^).

**Table 1:** The Clinical and biochemical/ hormonal data in women with polycistic ovary syndrome (PCOS) patients and healthy controls

**Variable**	**Women with PCOS (n_40)**	**Healthy controls (n_43)**	**p**
Age, years	22.97 ± 5.18	23.63 ± 4.60	>0.05
BMI, kg/m2	24.40 ± 5.82	25.44 ± 4.82	>0.05
Waist/hip ratio	0.84 ± 0.78	0.83 ± 0.73	>0.05
Fasting insulin, μ IU/ml	14.92 ± 9.96	9.25 ± 7.90	<0.01*
HOMA-IR	3.98(1.4-7.9)	1.91(0.74-4.84)	<0.01*
Total cholesterol, mg/dl	179.63 ± 26.62	151.46 ± 26.74	<0.01*
Triglyceride, mg/dl	109.75 ± 54.92	78.82 ± 32.55	<0.01*
LDL-C, mg/dl	99.45 ± 26.60	84.72 ± 23.51	<0.01*
FSH, m IU/ml	5.39 ± 1.82	5.88 ± 1.76	>0.05
LH, m IU/ml	5.79 ± 1.94	5.76 ± 2.44	>0.05
Estradiol, pg/ml	43.44 ± 22.32	72.43 ± 38.00	<0.01*
Free testosterone,pg/m	2.81 ± 1.02	1.44 ± 0.62	<0.01*
17-OHprogesterone,ng/ml	1.41 ± 0.55	0.90 ± 0.64	<0.01*
DHEAS, μq/dl	275.65 ± 115.45	195.67 ± 92.75	<0.01*
CIMT, mm	0.51 ± 0.052	0.42 ± 0.043	<0.01*
Copeptin, pmol/L	12.61 ± 3.05	9.60 ± 2.80	<0.01*

*p <0.05 was accepted as statistically significant.

*BMI* body mass index, *HOMA-IR* homeostasis model assessment insulin resistance index, *HDL-C* high density lipoprotein cholesterol, *LDL-C* LOW density lipoprotein cholesterol, *hs-CRP* high-sensitive C- reactive protein, *TSH* thyroid stimulating hormone, *FSH* follicle-stimulating hormone, *LH* luteinizing hormone, *DHEAS* dehydroepiandrosterone sulfate, *CIMT* carotid intima media thickness.

Mean fasting insulin, HOMA-IR, triglyceride, total cholesterol, LDL-C, free testosterone, total testosterone, 17 OH progesterone, DHEAS, CIMT levels were significantly higher and estradiol were significantly lower in PCOS patients (p < 0.05) (Table 1).

**Figure 1:**
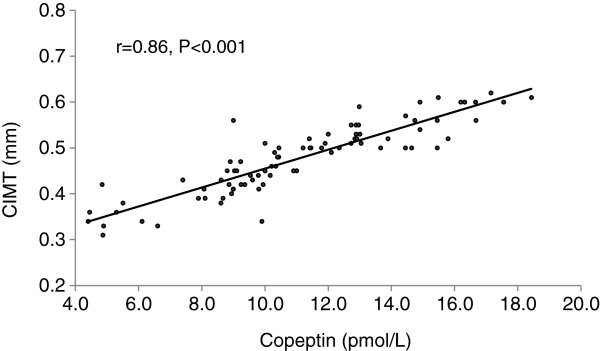
Linear correlation between copeptin and carotis intima media thickness (CIMT).

Mean copeptin level was in 12.61 ± 3.05 pmol/L in PCOS patients while mean copeptin level was 9.60 ± 2.80 pmol/L in healthy control women (p < 0.001). After adjustment for age and BMI, copeptin level was positive correlated with fasting insulin, TG, free testosterone levels, CIMT, HOM A-IR, FG score and negative correlated with HDL- C and estradiol levels (Table 2). In multiple linear regression analyses copeptin was found to be significantly associated with CIMT (beta coefficient = 0.86, p = 0.002) (age, BMI were included in the model). The correlation between copeptin and CIMT was shown in Figure 1.

**Table 2:** Correlation of age and body mass index adjusted copeptin levels with cardio-metabolic and endocrinologic parameters

**Variable**	**r**	**P**
Waist/hip ratio	0.11	0.253
Fasting glucose	0.10	0.533
Fasting insulin	0.41	<0.001*
HOMA	0.47	<0.01*
TC	-0.06	0.368
TG	0.01	0.470
HDL-C	*-0.35*	<0.01*
LDL-C	0.10	0.290
hsCRP	0.04	0.422
CIMT	*0.86*	<0.001*
Cortisol	-0.06	0.384
Estradiol	-0.24	0.02*
17 OH-progesterone	0.15	0.181
ACTH	-0.02	0.457
Free testosterone	*0.25*	0.02*
DHEA	0.06	0.359
FG score	*0.38*	<0.001*

*p <0.05 was accepted as statistically significant.

ACTH: adrenocorticotropic hormone, CIMT: carotid intima-media thickness, CRP: C-reactive protein, DHEA: dehydroepiandrosterone, FG score: Ferriman–Gallwey score, HDL-C: high-density lipoprotein cholesterol, HOMA: homeostasis model assessment, LDL-C: low-density lipoprotein cholesterol, R: Pearson linear correlation coefficient.

Mean CIMT level was 0.51 ± 0.052 millimeter in PCOS patients while mean CIMT level was 0.42 ± 0.043 millimeter in healthy control women (p < 0.01). A significant positive correlation was found between copeptin, fasting insulin, triglyceride, free testosterone , HOMA-IR and CIMT measurement (Table 3).

**Table 3:** Correlation of carotid intima media thickness with cardiometabolic parameters

**Variable**	**R**	**P**
Waist/hip ratio	0.18	0.141
Fasting glucose	0.02	0.450
Fasting insulin	0.28	0.02*
HOMA	0.29	0.02*
TG	0.31	0.01*
TC	0.11	0.254
HDL-C	*-0.35*	0.013*
LDL-C	0.08	0.327
hsCRP	0.3	<0.01*
Free testeron	0.28	0.03*
Copeptin	0.39	<0.001*
FG score	0.26	0.04*

*p <0.05 was accepted as statistically significant.

CIMT: carotid intima-media thickness, hsCRP: high sensitivity C-reactive protein, FG score: Ferriman–Gallwey score, HDL-C: high-density lipoprotein cholesterol, HOMA: homeostasis model assessment, LDL-C: low-density lipoprotein cholesterol, R: Pearson linear correlation coefficient.

The patient and control group examined separately and the correlations between copeptin and cardio-metabolic parameters were not different for healthy and PCOS group.

## Discussion

The present study confirms that serum levels of copeptin are increased in patients with PCOS, and, for the first time to our knowledge, demonstrates that elevated circulating serum levels of copeptin may provide important prognostic information in patients with PCOS. Copeptin showed significant correlations with cardiometabolic parameters in dependent of age and obesity.

PCOS women represent an intriguing biological model illustrating the relationship between hormonal pattern and cardiovascular risk profile, presenting a cluster of cardiovascular features, such as obesity, insulin resistance, hypertension, impaired cardiopulmonary functional capacity, autonomic dysfunction and low-grade chronic inflammation [18]. In recent substudy of the Women’s Ischemia Evaluation Study (WISE) [15] shown that women with PCOS have a larger number of cardiovascular events. In this study, CVD was positively correlated with free testosterone. In addition, the event free survival (including fatal and non fatal events) was significantly lower in PCOS compared with non-PCOS women. In our study cardiometabolic parameters including HOM A-IR, TG, LDL-C, free testosterone, DHEAS, CIM T were significantly higher in PCOS patients and positively correlated with copeptin levels. Also, FG that reflects androgen effects was positively correlated with copeptin and CIM T.

Orio F jr et al. [19] examined women with PCOS have significantly elevated PAI-1 activity independent of obesity. In Victor et al. [20] study an association was found between insulin resistance and an impaired endothelial and mitochondrial oxidative metabolism. They concluded that the inflammatory state related to insulin resistance in PCOS affects endothelial function. In presented study hsCRP and insulin resistance were found positive correlated with CIMT consistent with this hypothesis.

Copeptin, the C-terminal portion of provasopressin, is a glycosylated polypeptide comprising 39 amino acids and harboring a leucine-rich core segment [21]. It is a neurohormon (NH) of the AVP system [22] that is co-secreted with AVP from hypothalamus. It has also been termed AVP-associated glycopeptide, and was initially described by Holwerda in 1972 [21,23]. However, copeptin has recently come into clinical practice, and has been regarded as a novel NH. Recent studies showed that copeptin was elevated in acute myocardial infarction (AMI) and resulted in better diagnostic performance when assessed in combination with cardiac troponin, particularly during the first hour after onset of symptoms [24-26]. A negative test for both copeptin and troponin resulted in a remarkably high negative predictive value, that was helpful for a rapid rule out of AMI [24]. Furthermore, copeptin seems to have prognostic implications in patients with severe disorders such as severe congestive heart failure and patients with cardiac failure after AMI [27,28]. In patients with stable angina pectoris, copeptin showed a higher prognostic power regarding the endpoints death, stroke, or myocardial infarction than troponin [29]. Previous studies have shown that copeptin is not only a marker of cardiovascular diseases, but of other conditions as well. Potential links of copeptin with DM, metabolic syndrome (MetS) and microalbuminuria have drawn particular interest in the recent years. The AVP system has also been suggested to contribute to insulin resistance and DM potentially through a variety of mechanisms including stimulation of glucagon and ACTH secretion and glycogenolysis, etc. [30]. Therefore, copeptin, as a surrogate marker of this system, might also be associated with disrupted glucose homeostasis: a recent study demonstrated that increased copeptin levels were found to be associated with prevalent DM at baseline (p = 0.04) and insulin resistance (p < 0.001) in a large population of 4742 subjects (cross-sectionally) [30]. Consistent with this, copeptin was also reported to have a cross-sectional association with MetS in a large population of subjects [31]. In summary, based on the recent studies [30-32], copeptin may also be regarded as a promising marker of cardiometabolic risk (beyond established predictors of future cardiometabolic disease including fasting glucose, etc.) may serve as an additional guide in the early identification and management of subjects at risk for these conditions. However, in PCOS patients copeptin level has not been evaluated, yet. In our study we evaluated copeptin level in PCOS patients and we observed PCOS patients had higher copeptin levels. Additionally, we obtained positive correlation between copeptin and CIMT.

Copeptin may have a predictive role for detecting cardiometabolic risk in potential diseases. Therefore copeptin seems to be a marker that will enable the detection of cardiac injury in advancing age PCOS patients at an early stage. Copeptin appeared to have an important role in metabolic response and subsequent development of atherosclerosis in insulin resistant, hyperandrogenemic PCOS patients.

## Competing interests

The authors declare that they have no competing interests.

## Authors’ contributions

BK: have made contributions to conception and design, acquisition of data, and analysis and interpretation of data. MO: have made contributions to conception and design, acquisition of data, and analysis and interpretation of data MK, OT: have made contributions to conception and design , acquisition of data, and analysis and interpretation of data. EC, NCB, MSA: have made contributions to acquisition of data. TD: have made contributions to conception, design and interpretation of data. All authors read and approved the final manuscript.
